# Simple Models to Study Spectral Properties of Microbial and Animal Rhodopsins: Evaluation of the Electrostatic Effect of Charged and Polar Residues on the First Absorption Band Maxima

**DOI:** 10.3390/ijms22063029

**Published:** 2021-03-16

**Authors:** Andrey A. Shtyrov, Dmitrii M. Nikolaev, Vladimir N. Mironov, Andrey V. Vasin, Maxim S. Panov, Yuri S. Tveryanovich, Mikhail N. Ryazantsev

**Affiliations:** 1 Nanotechnology Research and Education Centre RAS, Saint Petersburg Academic University, 8/3 Khlopina Street, 194021 St. Petersburg, Russia; andriei.shtyrov@gmail.com (A.A.S.); dmitrii.m.nikolaev@gmail.com (D.M.N.); vova_mironov_97@mail.ru (V.N.M.); 2 Institute of Chemistry, Saint Petersburg State University, 7/9 Universitetskaya Nab., 199034 St. Petersburg, Russia; m.s.panov@spbu.ru (M.S.P.); tys@bk.ru (Y.S.T.); 3 Institute of Biomedical Systems and Botechnologies, Peter the Great St. Petersburg Polytechnic University, 29 Polytechnicheskaya Street, 195251 St. Petersburg, Russia; vasin_av@spbstu.ru

**Keywords:** rhodopsins, spectral properties of rhodopsins, spectral tuning in rhodopsins, engineering of red-shifted rhodopsins, photobiology, biological photosensors

## Abstract

A typical feature of proteins from the rhodopsin family is the sensitivity of their absorption band maximum to protein amino acid composition. For this reason, studies of these proteins often require methodologies that determine spectral shift caused by amino acid substitutions. Generally, quantum mechanics/molecular mechanics models allow for the calculation of a substitution-induced spectral shift with high accuracy, but their application is not always easy and requires special knowledge. In the present study, we propose simple models that allow us to estimate the direct effect of a charged or polar residue substitution without extensive calculations using only rhodopsin three-dimensional structure and plots or tables that are provided in this article. The models are based on absorption maximum values calculated at the SORCI+Q level of theory for cis- and trans-forms of retinal protonated Schiff base in an external electrostatic field of charges and dipoles. Each value corresponds to a certain position of a charged or polar residue relative to the retinal chromophore. The proposed approach was evaluated against an example set consisting of twelve bovine rhodopsin and sodium pumping rhodopsin mutants. The limits of the applicability of the models are also discussed. The results of our study can be useful for the interpretation of experimental data and for the rational design of rhodopsins with required spectral properties.

## 1. Introduction

Rhodopsins are photosensitive membrane proteins that have been discovered in many species across all three life domains. The natural diversity of the rhodopsins’ first absorption band maxima ($\lambda_{max}$) is achieved via the variation of the proteins’ amino acid compositions during evolutionary processes [1,2]. The same strategy is used in modern technologies to obtain rhodopsin variants with an optimal $\lambda_{max}$ [3,4,5,6]. In this context, it is desirable to develop methodologies for prediction of the $\lambda_{max}$ change caused by the modifications of the primary protein structure, e.g., single or multiple amino acid substitutions ($\Delta \lambda_{max}$).

Site-directed mutagenesis is a common experimental technique that allows for $\Delta \lambda_{max}$ evaluation. In many studies, amino acid substitutions are introduced into rhodopsins to measure $\Delta \lambda_{max}$ and, consequently, to estimate the substituted residue contribution to the absorption maximum [7,8,9]. The objectives of these studies are to evaluate the correlations between the type/position of a rhodopsin residue and the contribution of this residue to $\lambda_{max}$ and, ultimately, to establish general rules that allow for controlling $\lambda_{max}$. However, the interpretation of the measured $\Delta \lambda_{max}$ is not straightforward. Generally, a substitution of a single residue can lead to reorganization of the protein internal H-bond network changing positions of other residues and, therefore, their impact on the spectral properties. For such substitutions, a measured $\Delta \lambda_{max}$ cannot be attributed exclusively to the mutated residue, and the aforementioned effects must be taken into account. Apparently, this indirect spectral tuning due to reorganization of the internal H-bond network is common for rhodopsins. For example, such H-bond network reorganization is responsible for the origin of the spectral shift between anion-free and chloride-ion-bound forms of halorhodopsin from *Natronomonas pharaonis* [10], evolutionary switch between ultraviolet and violet vision in vertebrates [11], and between visual rhodopsins from *Alloteuthis subulata* and *Loligo forbesii* squids [12].

In addition to experimental studies, computational modeling can be involved. In general, computational models enable not only the calculation of $\Delta \lambda_{max}$ but also the evaluation of its direct and indirect parts. Currently, hybrid quantum mechanics/molecular mechanics (QM/MM) models are able to reproduce experimental $\lambda_{max}$ and $\Delta \lambda_{max}$ values with good accuracy (within 20–30 nm and just a few nm from experiment, respectively), assuming that a high-quality three-dimensional protein structure is provided [11,13,14,15,16,17,18]. However, evaluation of QM/MM $\Delta \lambda_{max}$ values is computationally expensive and not always easy. Thus, alternative less-demanding models are desirable for the interpretation of experimental data and initial rational design. These models can be less general and rigorous than QM/MM models, but they should allow for fast and simple $\Delta \lambda_{max}$ prediction.

Here, for visual and microbial rhodopsins, we proposed such simple models that allow us to estimate the direct electrostatic part of $\Delta \lambda_{max}$ for charged/polar amino acid substitution. The models are based on the precalculated high-level ab initio data. Application of these models requires only three-dimensional structures of rhodopsins, i.e., either X-ray structures or structures generated by comparative modeling. As a test, these models were applied to estimate the direct part of $\Delta \lambda_{max}$ for charged and polar residue substitutions in bovine rhodopsin and sodium pumping rhodopsin KR2. The obtained data were validated both against more sophisticated ab initio QM/MM calculations and against experiment.

## 2. Results

The results are described in the following sequence. First, the major principles of spectral tuning that make the proposed models possible are described. Then, models are introduced and applied to mutants of bovine rhodopsin and sodium pumping rhodopsin as an example. Finally, the limits of applicability of these models are discussed

### 2.1. Steric and Electrostatic Factors in Rhodopsin Spectral Tuning

The tuning of the rhodopsins’ first spectral band has been widely investigated [13,16,17,19,20]. Two factors are found to be the most important: the steric and electrostatic interactions of PSBs with surrounding amino acids of the opsin. Several other factors, such as the polarization of the retinal environment or inter-residual charge transfer within the binding pocket, have also been studied but were found to be less significant [21,22].

A substantial modification of the steric interaction between the protein pocket and the chromophore can lead to the distortion of the chromophore and, consequently, to a change in $\lambda_{max}$. If this is the case, $\Delta \lambda_{max}$ evaluation requires detailed information about the chromophore geometrical parameters. Generally, the resolution of available X-ray structures is not good enough to obtain these parameters with required precision. To date, the geometrical parameters of sufficient quality can be obtained only from high-level computational models, and simpler approaches are hardly possible. To cause prominent change in the steric interaction of PSB with opsin, one has to either introduce/remove a bulky residue in the protein binding pocket or to introduce/remove a distant residue that causes a substantial deformation of the binding pocket. Although modification of steric interactions for $\lambda_{max}$ tuning occurs in nature [23], rational design of rhodopsins with specific $\lambda_{max}$ by adjustment of steric interactions is not straightforward.

On the contrary, the electrostatic tuning mechanism is not only quite common in nature but also can be more easily utilized for rational design. Due to the charge transfer character of PSB $S_{0}\to S_{1}$ transition [24,25], $\lambda_{max}$ is very sensitive to the electrostatic field generated by the amino acids constituting an opsin. The contribution of any residue to this electrostatic field is primarily determined by its charge or dipole moment and its position relative to the chromophore. The contributions of quadrupoles and the multipoles of higher order can be neglected [26]. Generally, amino acid substitution can change $\Delta \lambda_{max}$ in two ways: either directly or indirectly. In the first case, $\Delta \lambda_{max}$ is obtained via substitution of the original amino acid by an amino acid with different charge/dipole moment. In the second case, $\Delta \lambda_{max}$ is caused by a substitution that induces reorganization of the whole protein, including charged and polar residues and changing the electrostatic field in the chromophore region.

**Figure 1:**
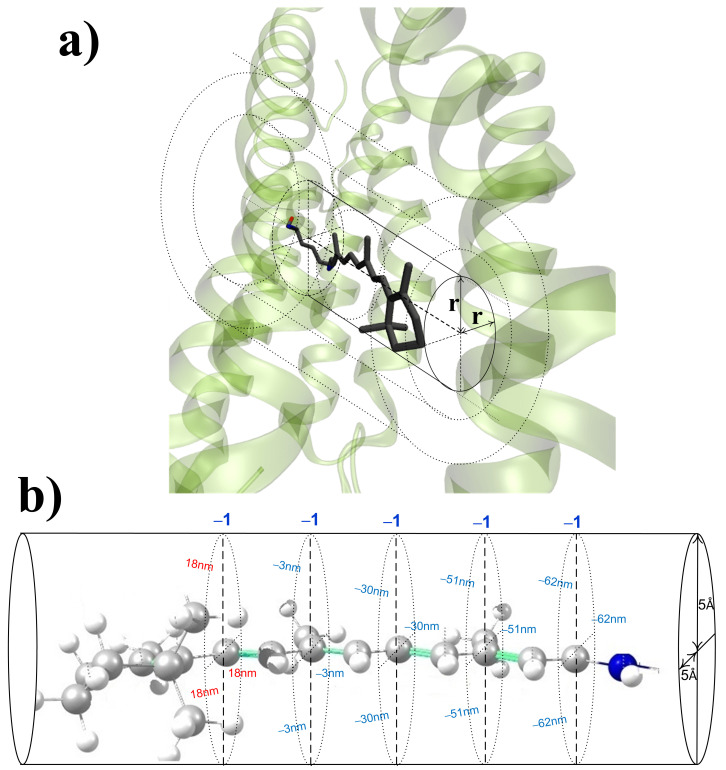
(**a**) Illustration of the “cylindrical” symmetry assumption for the protonated Schiff base (PSB). The impact of a charged/polar residue on $\lambda_{max}$ depends only on its charge/dipole moment and its distance to/orientation along the chromophore axis. (**b**) Spectral shift values caused by a unit negative charge (denoted as ‘−1’) located at 5 Å from different reference atoms of all-trans PSB. Negative spectral shift values (reference atoms C15, C13, C11, C9) are presented in blue; positive spectral shift value (reference atom C7) is presented in red.

Unlike the steric part of $\Delta \lambda_{max}$, the electrostatic part can be treated by a simple, practical model. To make it possible, the following assumptions must be done:

1. $\lambda_{max}$ can be evaluated as the vertical excitation energy of one characteristic snapshot that is close to the Gibbs free energy minimum of the whole protein. Protein dynamics can modify absorption band counters, but it does not affect the position of its maximum significantly.
2. The impact of a charged residue on $\lambda_{max}$ is equal to the impact of a unit negative/positive charge located at the center of the charged group of this residue;
3. The impact of a polar residue to $\lambda_{max}$ is equal to the impact of a dipole located at the center of the polar group of the residue.
4. If substantial reorganization of the H-bond network does not occur, the impact of each residue on $\lambda_{max}$ can be treated independently from the rest of the residues; i.e., we assume that all impacts are additive.
5. The impact of a charged/polar residue on $\lambda_{max}$ depends only on its charge/dipole moment and its distance to/orientation along the chromophore axis (see Figure 1). For charges, this “cylindrical” symmetry allows for reducing a four-dimensional function $\Delta \lambda_{max}$ = *f* (three Cartesian coordinates for a charge location) to a simpler three-dimensional function $\Delta \lambda_{max}$ = *f*(two Cartesian coordinates for a charge location). For polar residues, an additional argument, which describes the orientation of the dipole moment relative to the chromophore axis, should be added.
6. Although the electrostatic field always modifies a chromophore geometry by alternate changing of the length of double and single bonds, the effect of this geometry change on $\Delta \lambda_{max}$ can be neglected.

The first point is the widely used approximation for rhodopsin $\lambda_{max}$ modeling. Although more molecular dynamics studies are necessary to understand the limits of applicability for this assumption, inhomogeneous broadening of the absorption band is not yet reported for rhodopsins in contrast to some other photosensitive proteins [27,28]. Moreover, static QM/MM models have already been tested intensively and proven to be able to reproduce experimental $\lambda_{max}$ for dozens of rhodopsins [10,12,13,16,29,30]. It is worth mentioning that a system of interest can consist of a mixture of two or more stable forms, such as 13-cis and all-trans retinal containing the ground state of bacteriorhodopsin or different protonation states of titratable residues in Anabaena sensory rhodopsin [31]. If this is the case, several representative snapshots should be taken into account. Points 2 and 3 are the common coarse-grained approximation with well-known limitations [32,33]. For the last three points, additional justifications and discussion is given in the section “Limitations of the proposed models” and in the Supplementary Materials to avoid readers’ distraction from the main subject.

If the statements above are assumed, a two-dimensional grid for charges and a three-dimensional grid for dipoles can be calculated only once with a robust quantum mechanical method, and, then, one can use graphical representations, tables, or fitting by suitable functions for fast data acquisition. An approximate evaluation of $\Delta \lambda_{max}$ using these tabulated data can be performed only based on the knowledge of charges/dipoles of altered residues and their positions relative to the chromophore. In other words, all required information can be obtained from an X-ray structure or a structure predicted by comparative modeling.

### 2.2. Models to Evaluate the Direct Electrostatic Effect of Charged Residues

**Figure 2:**
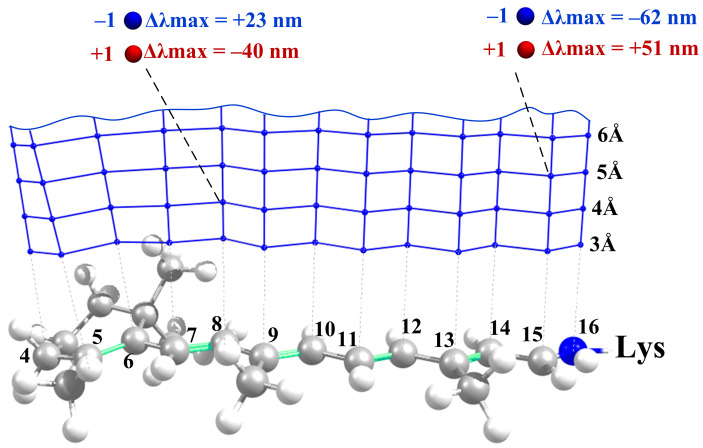
Grid representing the positions of a unit charge relative to the all-trans PSB. At each grid point we performed an ab initio calculation of $\Delta \lambda_{max}$ values for the positive and negative unit charges.

For each chromophore and negative/positive charges, we derived the numerical function that relates the position of a charged residue to the chromophore and its impact on $\lambda_{max}$ (Figure 2).

The geometries of the chromophores were kept as calculated in the gas phase, i.e., without external charges. We constructed a grid for the placement of unit charges as follows: We plotted thirteen grid lines from thirteen PSB reference atoms perpendicular to the chromophore axis (see Figure 2). Along each grid line, we placed unit charges at the fixed distances from the retinal chromophore, from 3 Å to 18 Å with the 1 Å interval (16 points along each grid line). In total, we performed 13 × 16 = 208 calculations for each charge and for each chromophore.

**Figure 3:**
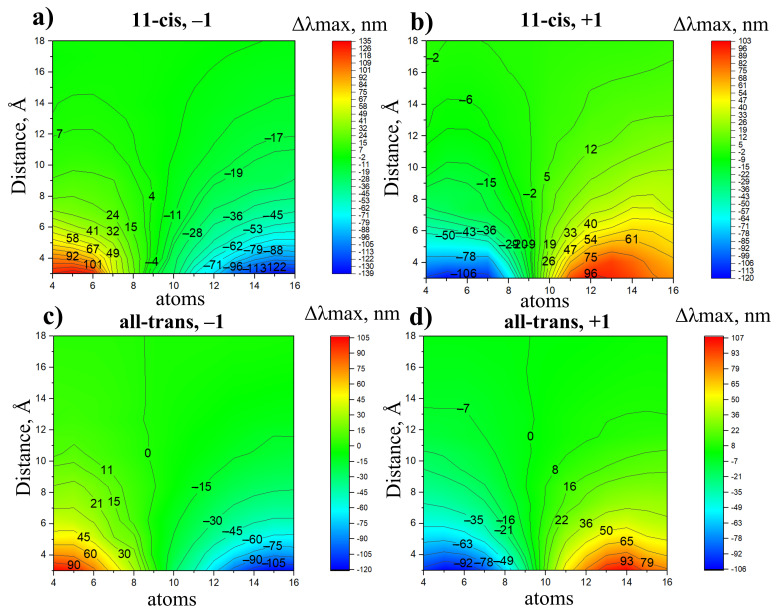
The impact of a unit charge on PSB $\lambda_{max}$ as a 2D function of its position relative to the chromophore. (**a**) 11-cis PSB, negative charge; (**b**) 11-cis PSB, positive charge; (**c**) all-trans PSB, negative charge; (**d**) all-trans PSB, positive charge

The calculated reference absorption maxima for the 11-cis and all-trans PSB without external charges $\lambda_{max}^{ref}$ were found to be 595 nm and 596 nm, respectively. For each chromophore and charge type and position, we performed the SORCI+Q calculation of the absorption maximum value ($\lambda_{max}^{i}$). Then, we derived the effect of the charged residue placed at a certain position relative to the retinal chromophore as $\Delta \lambda_{max}^{i}=\lambda_{max}^{i}-\lambda_{max}^{ref}$. The results of these calculations are illustrated as 2D functions $\Delta \lambda_{max}^{i}$ (reference atom, distance) in Figure 3, Figure S1 and Tables S1–S4.

The four panels in Figure 3 correspond to the four considered systems: (a) a negative charge, 11-cis chromophore; (b) a positive charge, 11-cis chromophore; (c) a negative charge, all-trans chromophore; (d) a positive charge, all-trans chromophore. These 2D functions allow us to estimate $\Delta \lambda_{max}$ caused by the charged residue placed at a certain position in the rhodopsin. For example, a positively charged residue (lysine, arginine, or protonated histidine) placed at 7 Å from the C${}_{14}$ PSB atom would cause an approximately +40 nm red shift for the 11-cis chromophore and a +36 nm red shift for the all-trans chromophore. On the other hand, a negatively charged residue (glutamic or aspartic acid) at the same position would cause an approximately −40 nm blue shift for 11-cis chromophore and a −35 nm red shift for the all-trans chromophore.

Several well-known rules/patterns can also be clearly seen from the plots in Figure 3:

1. The effect of a charged residue on $\lambda_{max}$ depends on (a) the sign of the charge and (b) the distance from the charge to the closest atom of the retinal.
2. A negative charge located in the NH region causes a blue shift; a negative charge located in the β-ionone ring region causes a red shift.
3. On the contrary, a positive charge located in the NH region causes a red shift; a positive charge located in the β-ionone ring region causes a blue shift.
4. The charges that are closer to the ends of the chromophore (atoms N${}_{16}$, C${}_{15}$, C${}_{6}$, and C${}_{5}$) cause larger shifts, while the charges that are close to the middle of the chromophore (atoms C${}_{12}$, C${}_{11}$, and C${}_{10}$) cause smaller shifts.

The effect of charged residues on $\lambda_{max}$ is slightly larger for 11-cis PSB than for all-trans PSB (Figure 3). To rationalize this fact, we calculated the charge distributions in the ground and the first excited states of 11-cis and all-trans PSB. The calculations were performed at the CASSCF/6-31G* level of theory. We found that the portion of positive charge, which is translocated from the NH region to the beta-ionone ring region upon photoexcitation, is larger for 11-cis PSB (0.30) than for all-trans PSB (0.21).

### 2.3. Models to Evaluate the Direct Electrostatic Effect of Polar Residues

**Figure 4:**
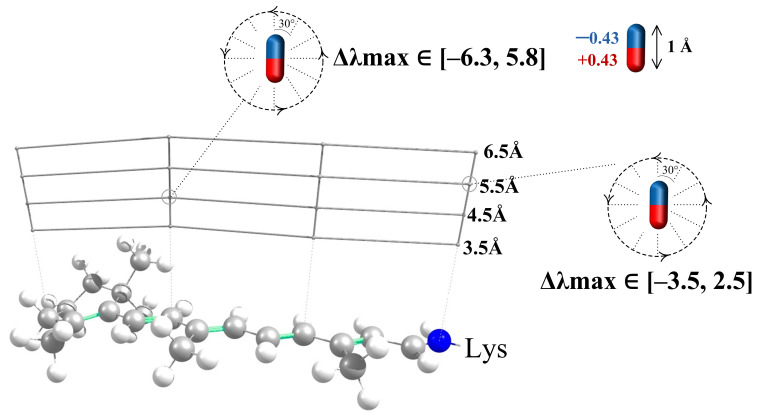
Grid representing the positions of a dipole moment relative to the all-trans PSB. At each grid point, we performed the ab initio calculation of $\Delta \lambda_{max}$ values for different orientations of the dipole moment relative to the chromophore axis.

**Figure 5:**
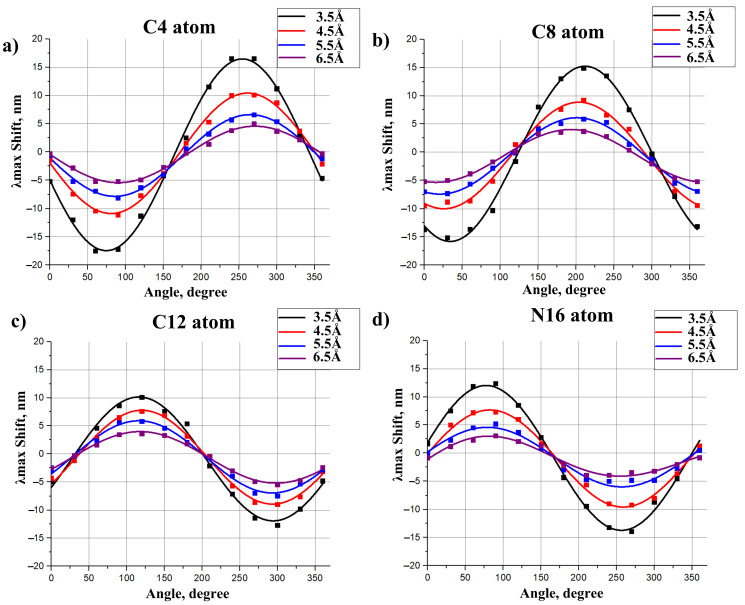
Impact of the dipole moment on $\lambda_{max}$ of the 11-cis PSB as a function of the angle between the dipole and the chromophore axis. Functions were calculated at different positions of the dipole relative to the chromophore. Dipoles were located at the distances 3.5 Å, 4.5 Å, 5.5 Å, 6.5 Å from the C${}_{4}$ (**a**), C${}_{8}$ (**b**), C${}_{12}$ (**c**), and N${}_{16}$ (**d**) chromophore atoms along the grid line perpendicular to the chromophore axis (see Figure 4). Dots represent the ab initio calculated values. Functions were derived as the spline interpolation of the calculated data.

**Figure 6:**
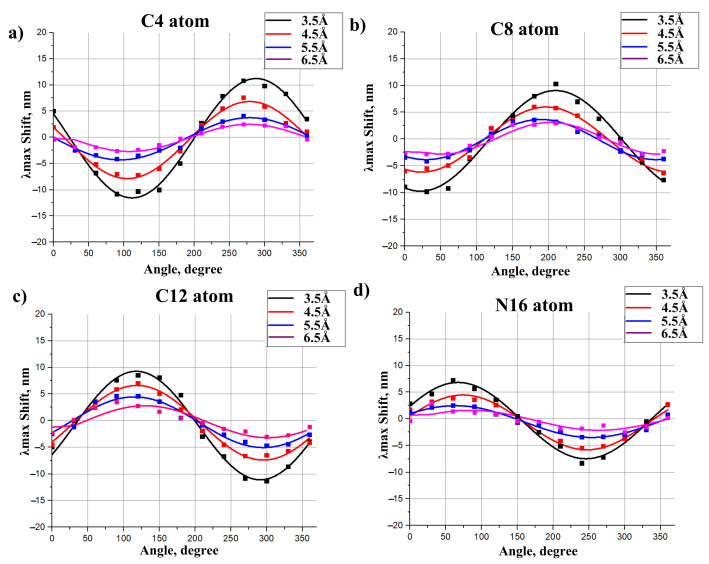
Impact of the dipole moment on $\lambda_{max}$ of the all-trans PSB as a function of the angle between the dipole and the chromophore axis. Functions were calculated for different positions of the dipole relative to the chromophore. Dipoles were located at the distances 3.5 Å, 4.5 Å, 5.5 Å, 6.5 Å from the C${}_{4}$ (**a**), C${}_{8}$ (**b**), C${}_{12}$ (**c**), and N${}_{16}$ (**d**) chromophore atoms along the grid line perpendicular to the chromophore axis (see Figure 4). Dots represent the ab initio calculated values. Functions were derived as the spline interpolation of the calculated data.

**Figure 7:**
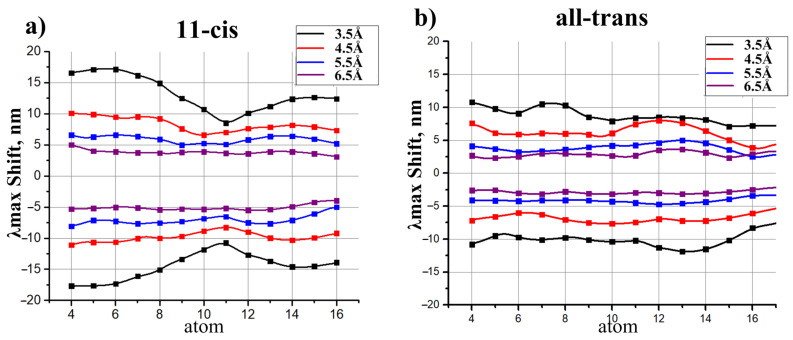
Largest possible negative and positive contributions of dipole moments to $\lambda_{max}$ of 11-cis PSB (**a**) and all-trans PSB (**b**) as functions of a dipole moment position along the chromophore axis. Functions were calculated at different distances of the dipole moment from the chromophore. Dots represent the ab initio calculated values. Functions were derived as the spline interpolation of the calculated data.

To derive models for polar residues, we followed the strategy similar to the approach used in the previous section. $\Delta \lambda_{max}^{i}$ caused by a polar residue depends not only on the distance of this residue to the chromophore but also on the orientation of this residue polar group (see Figure 4). Therefore, we calculated a numerical function that relates $\Delta \lambda_{max}^{i}$ and both the distances to the chromophore and the angle between a dipole and the chromophore axis. Each dipole was represented by two charges of a different sign (−0.43 and +0.43) that are situated 1.0 Å from each other (see Figure 4). The magnitudes of the charges and the distance between them were chosen in order to represent the Amber parameters for the -OH group of polar residues, such as Ser or Thr. The geometries of 11-cis PSB and all-trans PSB were kept as calculated in the gas phase, i.e., without an external electrostatic field. Four atoms of the 11-cis and all-trans chromophore (N${}_{16}$, C${}_{12}$, C${}_{8}$, C${}_{4}$) were taken as the reference atoms. We plotted four grid lines from these four reference atoms perpendicular to the chromophore (see Figure 4). Along each grid line we placed the center of the dipole at the fixed distances from the retinal chromophore, from 3.5 Å to 6.5 Å with the 1 Å interval (4 points along each grid line in total). The orientation of the dipole was varied by changing the angle γ that is defined as the angle between the chromophore axis and the line connecting oxygen and hydrogen atoms. γ varied from 0${}^{\circ }$ to 360${}^{\circ }$ with the step of 30${}^{\circ }$. The results of our calculations are presented in Figure 5 and Figure 6, Tables S5 and S6. We also performed spline interpolation of the calculated data to plot the largest possible negative and positive contributions of polar residues to $\lambda_{max}$ as functions of a dipole moment position along the chromophore axis (Figure 7).

Several rules/patterns can be derived from the plots presented in Figure 5 and Figure 6.

1. The effect of polar residues located further than 6–7 Å from the PSB can be neglected.
2. The impact of a polar residue on $\lambda_{max}$ is determined not only by the distance from the polar group of the residue to a given atom of the chromophore, as it is for charged residues, but also by the orientation of a polar group relative to the chromophore.
3. $\Delta \lambda_{max}$ ranges from a negative value (for example, −9 nm for a dipole situated at 4.5 Å from an atom of 11-cis PSB) to zero and then to a positive value (+7.5 nm for this dipole). Therefore, to estimate the effect of a polar residue on the rhodopsin absorption maximum, accurate structural information is required.

The effect of polar residues on $\lambda_{max}$ is slightly larger for 11-cis PSB than for all-trans PSB. This fact can be rationalized by the difference in the portion of positive charge translocated from the NH region to the beta-ionone ring region upon photoexcitation, as discussed in the Section 2.2.

Only the rotation of a dipole moment in the grid plane (Figure 4) was considered. Due to the symmetry of the system, the dipole moment component that is perpendicular to the grid surface should have a negligible effect on $\lambda_{max}$. To prove this, we located the dipole moment at 3.5 Å from the N16 PSB atom and rotated it perpendicular to the grid surface; $\Delta \lambda_{max}$ values were calculated with a step of 30${}^{\circ }$. The calculated spectral shift values did not exceed 1.5 nm, which is within the limits of QM calculation error.

### 2.4. Application of the Proposed Models to Evaluate the Direct Effect of Amino Acid Substitutions

Using the protocol described in the Methods section, we applied the proposed models to evaluate the direct effect of twelve amino acid substitutions in bovine rhodopsin (Rh) and sodium pumping rhodopsin (KR2). For charged residues, the data presented in Figure 3 were used to obtain the correspondence between their position and the possible spectral shift. For polar residues, the data presented in Figure 5, Figure 6 and Figure 7 were used to evaluate the range of possible spectral shift values due to the lack of information about the orientation of a polar group relative to PSB. The exact orientation of a polar group could be defined only by constructing the corresponding protein QM/MM model. The estimated direct effect values were compared with the experimentally observed and QM/MM calculated spectral shift values.

**Figure 8:**
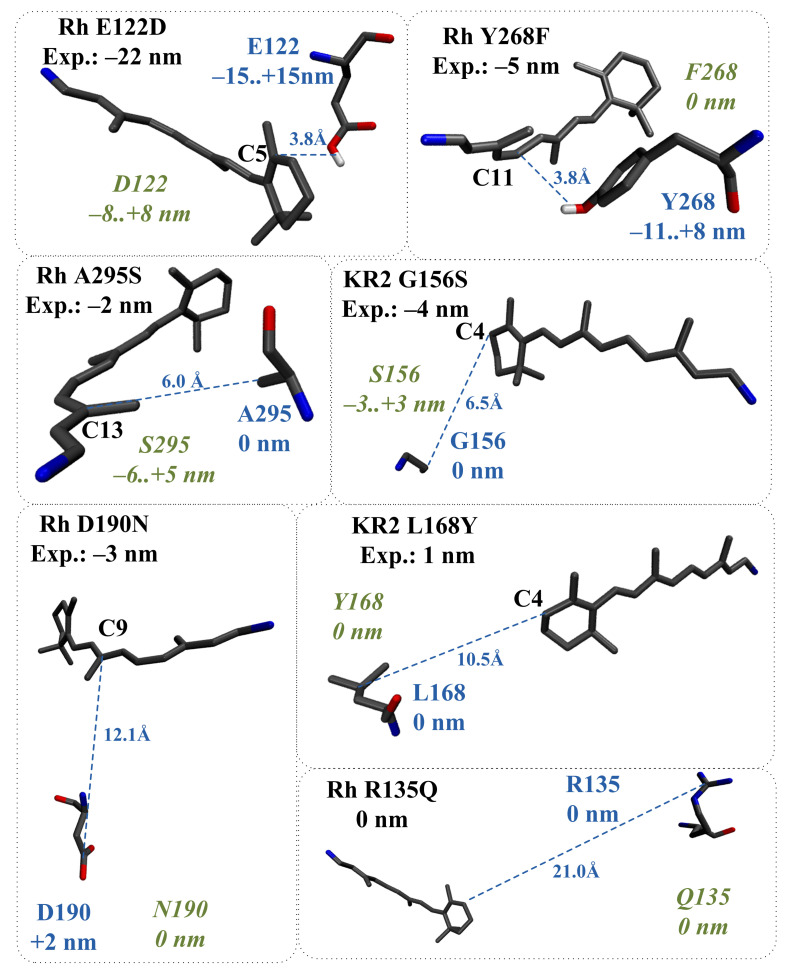
Bovine rhodopsin (Rh) and sodium pumping rhodopsin (KR2) mutants, for which the direct effect of amino acid substitution completely explains the experimentally observed spectral shift. Experimental spectral shift values are shown in black. The distances from the wild-type residues to the PSB and corresponding contributions to $\lambda_{max}$ are shown in blue. The evaluated contributions of new residues to $\lambda_{max}$ are shown in green. The distances are given in Å.

**Figure 9:**
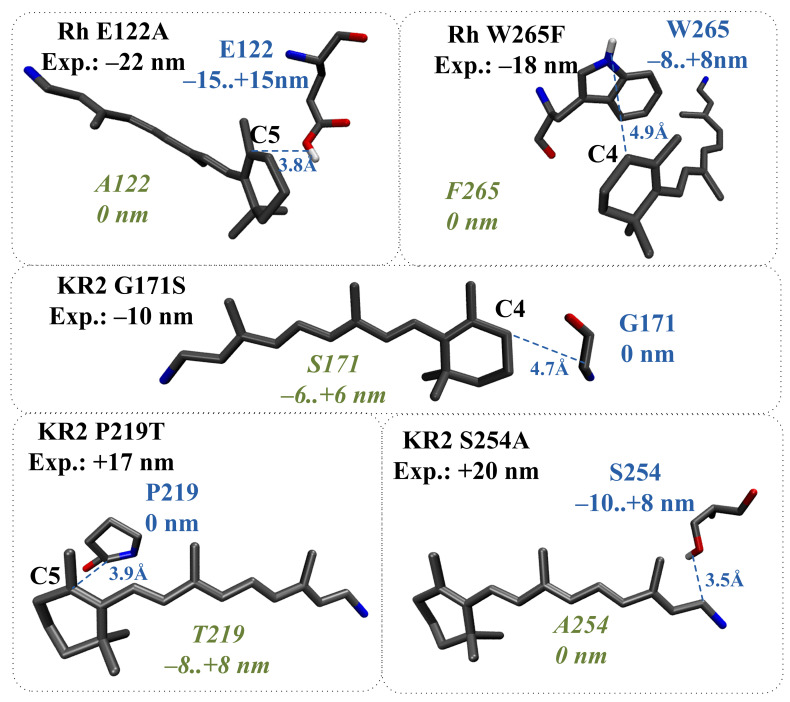
Bovine rhodopsin (Rh) and sodium pumping rhodopsin (KR2) mutants, for which the direct effect of amino acid substitution cannot completely explain the experimentally observed spectral shift. Experimental spectral shifts are shown in black. The distances from the wild-type residues to the PSB and corresponding contributions to $\lambda_{max}$ are shown in blue. The evaluated contributions of new residues to $\lambda_{max}$ are shown in green. The distances are given in Å.

**Figure 10:**
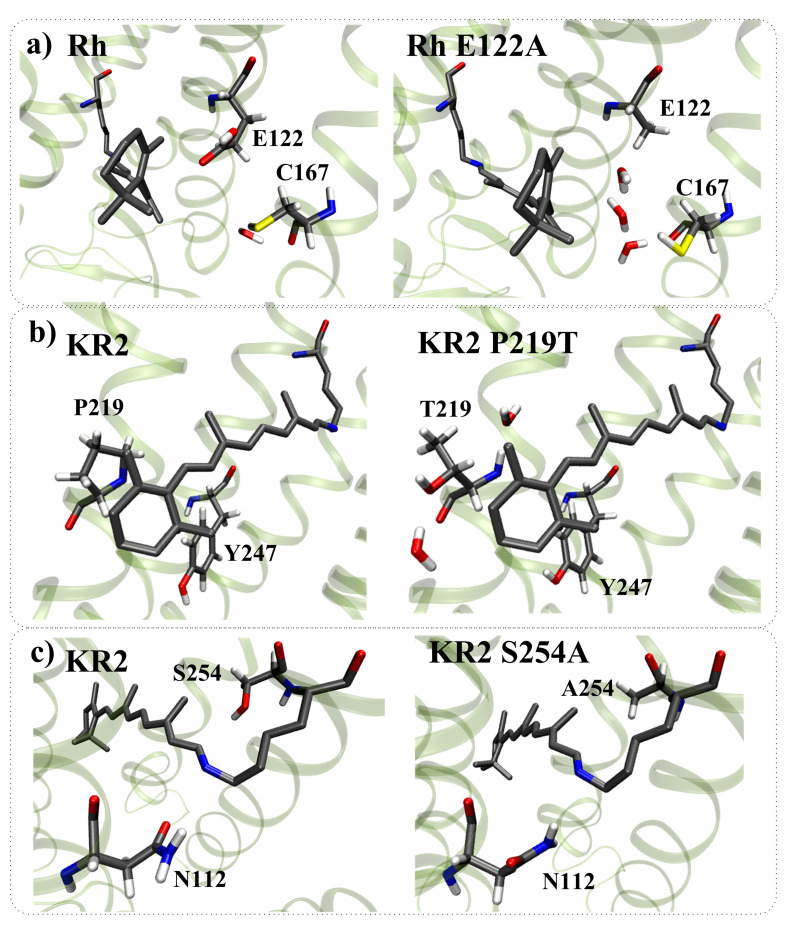
Structural reorganization caused by amino acid replacements. (**a**) E122A substitution in bovine rhodopsin (Rh); (**b**) P219T substitution in sodium pumping rhodopsin (KR2); (**c**) S254A substitution in sodium pumping rhodopsin (KR2).

According to the proposed models, for seven mutants, the direct effect of amino acid substitution completely explains the experimentally observed spectral shift (Figure 8 and green color-coding in Table 1). These estimations were also confirmed by the analysis of the corresponding QM/MM models. For five mutants, the direct effect of amino acid substitution cannot completely explain the experimentally observed spectral shift, and the indirect effect has to be taken into account (Figure 9 and brown color-coding in Table 1). The analysis of the corresponding QM/MM models confirmed that these five substitutions cause structural reorganization of the protein (Figure 10). Reorganization can include three components: (1) Reorientation of charged/polar residues in the protein due to the substitution; (2) Addition/deletion of water molecules. Water molecules possess a dipole moment and for this reason can impact on $\lambda_{max}$; (3) Change in the protonation state of titratable residues. Below, we describe the indirect effect of considered amino acid substitutions in more detail.

(1) The structural reorganization caused by E122A replacement in Rh (3.8 Å from C5 PSB atom) involves the reorientation of C167 residue and the addition of two water molecules located in the increased cavity at the substitution site (Figure 10a).
(2) The structural reorganization caused by P219T replacement in KR2 (3.9 Å from C5 PSB atom) involves the reorientation of the polar Y247 residue and the addition of two water molecules at the substitution site (Figure 10b).
(3) The structural reorganization caused by S254A replacement in KR2 (3.5 Å from C15 PSB atom) involves the reorientation of the polar N112 residue located in the vicinity of the N16 PSB atom (Figure 10c). The distance from the NH2 group of N112 to the N16 PSB atom decreases from 4.5 Å to 3.6 Å.
(4) The structural reorganization caused by G171S replacement in KR2 (4.7 Å from C4 PSB atom) involves the reorientation of the positively charged R246 residue located at 12 Å from C6 PSB in the wild-type protein. The charged center of R246 comes closer to beta-ionone part of PSB, leading to an additional slight blue shift. The water molecule located between G171 and the beta-ionone ring of PSB in wild-type KR2 moves away in the KR2 G171S mutant.
(5) The structural reorganization caused by W265F replacement in Rh (4.9 Å from C4 PSB atom) involves the reorientation of the polar Y191 residue and the addition of three water molecules in the increased cavity at the substitution site. According to our QM/MM model, W265F replacement has a non-negligible effect on retinal geometry. The spectral shift related to retinal geometry modification is −8 nm, while the experimentally observed spectral shift is −18 nm.

Overall, the proposed models can be applied not only to estimate the direct effect of amino acid substitution but also to determine if the indirect effect of amino acid substitution occurs.

### 2.5. Limitations of the Proposed Models

**Figure 11:**
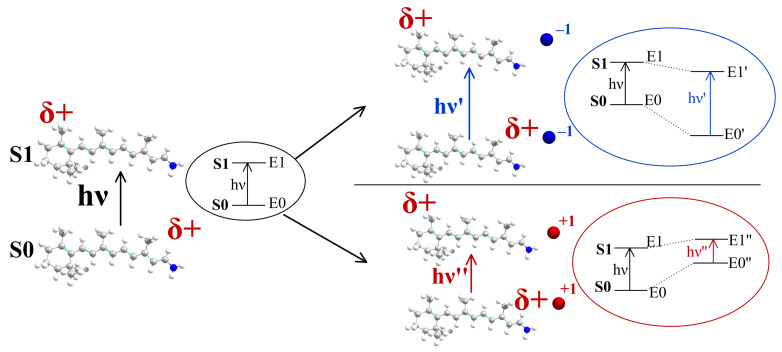
Difference in the charge distribution between $S_{0}$ and $S_{1}$ states of PSB leads to different electrostatic interactions of the chromophore with external charges.

As demonstrated in a number of experimental and computational studies [24,39,40,41,42], the positive charge located at the NH moiety of the chromophore after excitation partially relocates to the β-ionone ring moiety, making the NH part less positive and, accordingly, the β-ionone ring part more positive. This difference in the charge distribution between $S_{0}$ and $S_{1}$ states leads to different electrostatic interactions of the chromophore with external charges (see Figure 11).

The interaction between the charge density of the chromophore and the negative charge located in the NH region stabilizes the ground state more than the first excited state and, therefore, leads to a blue shift in the $S_{0}$ to $S_{1}$ band. On the other hand, a negative charge in the β-ionone region stabilizes the excited state more than the ground state, leading to a red shift. A positive charge in the NH region destabilizes the ground state more than the excited state, leading to a red shift. Finally, a positive charge in the β-ionone region destabilizes the excited state more than the ground state, leading to a blue shift (Figure 11).

**Figure 12:**
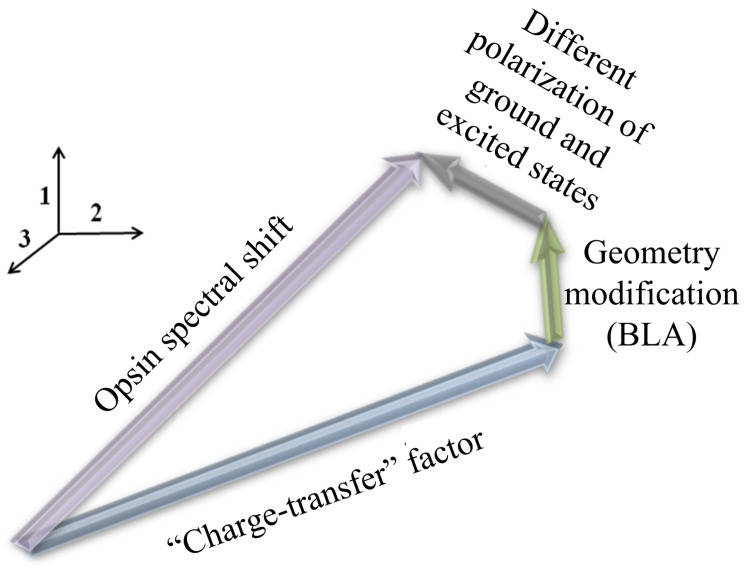
The electrostatic spectral tuning mechanism in rhodopsins involves three main factors: 1. The “charge transfer” factor related to difference in charge distributions of ground and excited states of the PSB. The positive charge partially translocates from the NH region to the β-ionone ring region upon photoexcitation. Therefore, ground and excited states of the chromophore possess different interactions with external charges. 2. The modification of the bond length alternation (BLA) of the chromophore by the external electrostatic field. 3. Differences in polarization of the ground and excited states of the PSB by the external electrostatic field.

If $\Delta \lambda_{max}$ was determined only by this “charge transfer” factor, and both $S_{0}$-to-$S_{1}$ charge redistribution and the geometry of the chromophore did not depend on an external electrostatic field, the impact of each residue on $\Delta \lambda_{max}$ would be independent of the rest of the residues; i.e., $\Delta \lambda_{max}$ of each residue would be additive. In fact, this additivity is broken due to the polarization effect caused by any charged or polar residue. The additional electrostatic field modifies both the magnitude of the $S_{0}\to S_{1}$ charge transfer due to different polarization of the ground and the excited states and the ground state geometry of the chromophore by changing the so-called bond length alternation (BLA), i.e., averaged difference between single- and double-bond lengths of the chromophore (Figure 12). However, as demonstrated, for example, for *N. Pharaonis* halorhodopsin [10], the contribution of these polarization effects to $\Delta \lambda_{max}$ is much smaller than the contribution of the “charge transfer” effect, and, in the absence of other protein residues reorganization, the $\Delta \lambda_{max}$ additivity can be considered as a good approximation.

The proposed models assume that the impact of a charged/polar residue on $\lambda_{max}$ depends only on its charge/dipole moment and its distance to/orientation along the chromophore axis but not a radial angle. To confirm this “cylindrical symmetry” assumption, we performed an additional set of calculations rotating negative unit charges at 4 Å around the 11-cis chromophore axis. The results confirm that calculated $\Delta \lambda_{max}$ only slightly depend on radial angles (See Supplementary Table S7 for details).

Finally, the proposed models do not take into account the possible distortion of the chromophore due to the steric interactions caused by amino acid substitution [23]. This effect can be accurately taken into account only by QM/MM models.

## 3. Materials and Methods

### 3.1. Ab Initio-Based Models

Geometries of 11 -cis PSB (protonated Schiff base) and all-trans PSB were optimized at the B3LYP/6-31G* level of theory. Absorption maxima values were calculated at the SORCI(6,6)+Q/6-31G* level of theory. Electrostatic embedding scheme was used to include the effect of external charges. The calculations were performed with the ORCA program, version 3.0.3 [43].

### 3.2. Evaluation of Spectral Shifts Caused by Amino Acid Replacements

To evaluate $\Delta \lambda_{max}$ values caused by amino acid substitutions, the corresponding three-dimensional structures of the wild-type proteins were used. For bovine rhodopsin (Rh), the 2.2 Å X-ray structure was used, RCSB code 1U19 [44]; for sodium pumping *Krokinobacter eikastus* rhodopsin 2 (KR2), the 1.8 Å structure was used, RCSB code 6RF6 [45]. The distance from the substituted amino acid to the closest atom of the retinal chromophore was measured using visualizing software (VMD program, v.1.9.3) [46]. The pdb file of the X-ray structure was used without any preliminary modifications. When the position of the residue was defined, we used the figures and tables presented in the Results section and the Supporing Information to determine the correspondence between the position of the residue and the possible spectral shift.

### 3.3. QM/MM Models Construction

To generate QM/MM models of rhodopsin mutants, we started from the corresponding wild-type X-ray structures. The amino acid substitutions were inserted into the wild-type X-ray structures using the Mutate Model algorithm implemented in Modeller v.9.15 program package [47]. The algorithm replaces the indicated amino acid in the protein X-ray structure and optimizes its position, leaving other protein residues intact. The retinal chromophore was inserted into the models and bound to the proper lysine residue (11-cis PSB, Lys296 for Rh; all-trans PSB, Lys255 for KR2). Afterward, models were hydrated with the Dowser++ algorithm [48]; the configuration parameters for running Dowser++ and the parameter set for the PSB were described in our previous work [29]. The PROPKA program, version 3.1 [49], was used to calculate the pKa values of titratable residues (pH = 7.0) and assign their protonation states; hydrogen atoms were added with the pdb2pqr program, version 2.1.1 [50]. The obtained models were optimized gradually first at the MM level (Amber96 force field [51], TIP3P for water) then at the QM/MM level utilizing the hybrid two-layer ONIOM (QM:MM-EE) scheme (QM = B3LYP/6-31G*; MM = AMBER96 for amino acids and ions, TIP3P for water, EE = electronic embedding). The ONIOM calculations were performed with Gaussian09 [52]. Fifty atoms of the retinal chromophore were included in the QM part; the link atom was placed at the N${}_{Z}$-C${}_{\epsilon }$ bond of Lys296. The SORCI+Q/6-31G* method was used to calculate the PSB absorption maxima values in the opsin environment represented as Amber96 point charges. The absorption maxima calculations were performed with the ORCA program, version 3.0.3 [43]. The reliability of the applied methodology for rhodopsins was tested in several previous studies [10,12,13,29,30].

## 4. Conclusions

The main goal of this article was to present a simple ab initio-based approach to evaluate $\Delta \lambda_{max}$ caused by substitution of charged or polar amino acids in visual and microbial rhodopsins. If the rhodopsin three-dimensional structure is available, $\Delta \lambda_{max}$ can be obtained from the plots and tables given in the article and Supplementary Materials. The performance of the proposed models is evaluated against a test set consisting of ten mutants of bovine and sodium pumping rhodopsins.

Additional, general conclusions of this study can be summarized as follows:

1. The contribution of charged residues to $\lambda_{max}$ strongly depends on their positions and varies from over 100 nm for counterions at the distance of around 3.5 Å from the nitrogen atom of the chromophore to several nm for the residues located at 18 Å.
2. The contribution of polar residues outside the binding pocket, i.e., more than 6–7 Å from the chromophore, is negligible.
3. The distance from a charged/polar residue to the closest atom of the chromophore is the main parameter that is required to estimate the contribution of this residue to $\lambda_{max}$. In addition, the information about the dipole moment orientation relative to the chromophore is important for the evaluation of contributions of polar residues.
4. An adequate model to evaluate $\lambda_{max}$ of a rhodopsin must take into account the effect of polar/charged residues in the binding pocket, i.e., within 6–7 Å, and the charged residues at least up to 16–18 Å. On one hand, these findings explain the success of “binding pocket models” [14,53], in which the main difference in $\lambda_{max}$ between two rhodopsins is attributed to the amino acid compositions of their binding pockets. On the other hand, these findings also reveal the limitations of the “binding pocket models” models, such as neglecting the charged residues beyond the binding pocket and the reorganization of polar/charged residues within the binding pocket due to distant amino acid substitutions.

The models proposed in this study can be used to estimate the direct part of $\Delta \lambda_{max}$ caused by residue substitution and, therefore, can be utilized both for the interpretation of experimental data and for the rational design of rhodopsins with specific spectral properties.

## Figures and Tables

**Table 1:** Spectral shifts in bovine rhodopsin (Rh) and sodium pumping rhodopsin (KR2) caused by amino acid substitutions. $\Delta \lambda_{max}^{exp}$—experimental spectral shift. $\Delta \lambda_{max}^{direct}$—the magnitude of the spectral shift estimated by the proposed models (Figure 3, Figure 5, Figure 6 and Figure 7 and Tables S1–S6). $\Delta \lambda_{max}^{QM/MM}$—the spectral shift calculated with the quantum mechanics/molecular mechanics models of rhodopsin mutants. Color coding: green—substitutions that do not induce structural reorganization (direct spectral tuning), brown—substitutions that cause a substantial structural reorganization (indirect spectral tuning).

Mutant	Type	$\Delta \lambda_{\max}^{\exp}$	$\Delta \lambda_{\max}^{\mathrm{direct}}$	$\Delta \lambda_{\max}^{\mathrm{QM}/\mathrm{MM}}$
Rh Y268F	polar/ nonpolar	−5 nm [34]	Y268: −11 to +8 nm 3.8 Å from C${}_{11}$ F268: 0 nm	−7 nm
Rh A295S	nonpolar/ polar	−2 nm [35]	A295: 0 nm S295: −6 to +5 nm 6.0 Å from C${}_{13}$	−7 nm
Rh D190N	charged/ polar	−3 nm [36]	D190: +2 nm 12.1 Å from C${}_{9}$ N190: 0 nm	+1 nm
Rh R135Q	charged/ polar	0 nm [37]	R295: 0 nm 21.0 Å from C${}_{4}$ Q135: 0 nm	−2 nm
KR2 L168Y	nonpolar/ polar	1 nm [38]	L168: 0 nm Y168: 0 nm 10.5 Å from C${}_{4}$	4 nm
Rh E122D	charged/ charged	−22 nm [34]	E122: +62 nm 4.8 Å from C${}_{5}$ D122: +38 nm E122D: −27 nm	−
Rh E122D	charged (protonated)/ charged (protonated)	−22 nm [34]	E122${}^{+}$: −15 to +15 nm 3.8 Å from C${}_{5}$ D122${}^{+}$: −8 to +8 nm E122D: −23 nm to 23 nm	−24 nm
KR2 G156S	nonpolar/ polar	−4 nm [38]	G156: 0 nm S156: −3 to +3 nm 6.5 Å from C${}_{4}$	−8 nm
KR2 G171S	nonpolar/ polar	−10 nm [38]	G171: 0 nm S171: −6 to +6 nm 4.7 Å from C${}_{4}$	−9 nm
Rh E122A	charged (protonated)/ neutral	−22 nm [34]	E122${}^{+}$: −15 to +15 nm 3.8 Å from C${}_{5}$ A122: 0 nm	−23 nm
Rh W265F	polar/ neutral	−18 nm [34]	W265: −8 to +8 nm 4.9 Å from C${}_{4}$ F265: 0 nm	−16 nm
KR2 S254A	polar neutral	+20 nm [38]	S254: -10 to +8 nm 3.5 Å from C${}_{15}$ A254: 0 nm	+24 nm
KR2 P219T	neutral/ polar	+17 nm [38]	P219: 0 nm T265: −8 to +8 nm 3.9 Å from C${}_{5}$	+12 nm

## Data Availability

The data that support the findings of this study are available from the corresponding author upon reasonable request.

## Supplementary Materials

Supplementary materials can be found at https://www.mdpi.com/1422-0067/22/6/3029/s1.
