# Supplementary material for: Simple Models to Study Spectral Properties of Microbial and Animal Rhodopsins: Evaluation of the Electrostatic Effect of Charged and Polar Residues on the First Absorption Band Maxima
Source: Int J Mol Sci. 2021 Mar 16;22(6):3029. doi: 10.3390/ijms22063029 (PMC8002287; doi:10.3390/ijms22063029)
Supplement: Supplementary file 1 [file ijms-22-03029-s001.pdf]

Supplementary Information for the manuscript
«Simple models to study spectral properties of microbial and animal rhodopsins: evaluation of the electrostatic effect of charged and polar residues on the first absorption band maxima»

Andrey A. Shtyrov, Dmitrii M. Nikolaev, Vladimir N. Mironov, Andrey V. Vasin, Maxim S. Panov, Yuri S. Tveryanovich, Mikhail N. Ryazantsev.

Figure S1. The effect of a unit negative charge on the absorption maximum of the protonated Schiff base as a function of the distance from the charge to the PSB reference atom.

a) Reference atom: C4

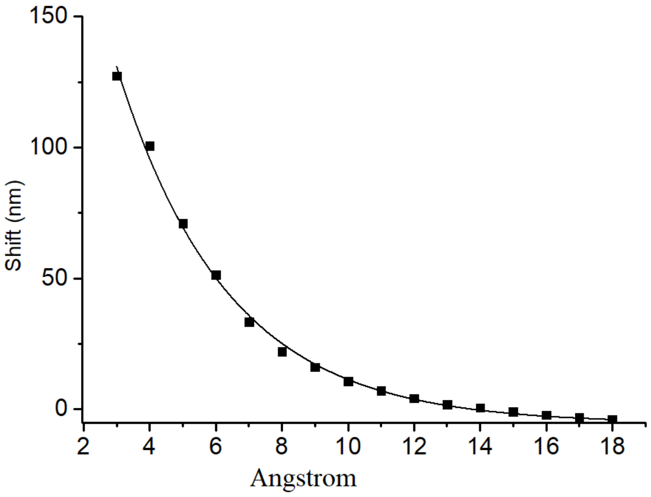

b) Reference atom C5

c)

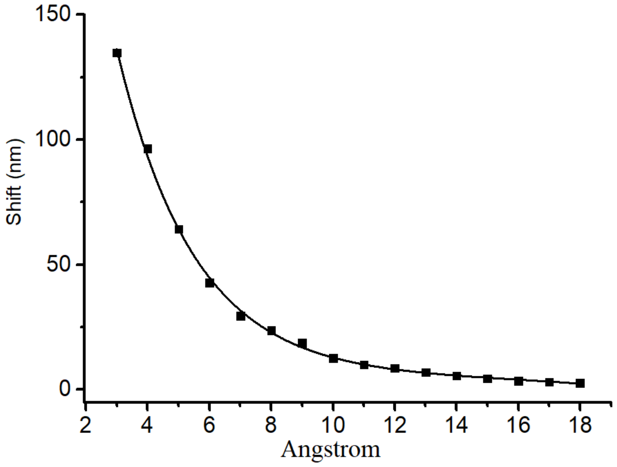

Reference atom C6

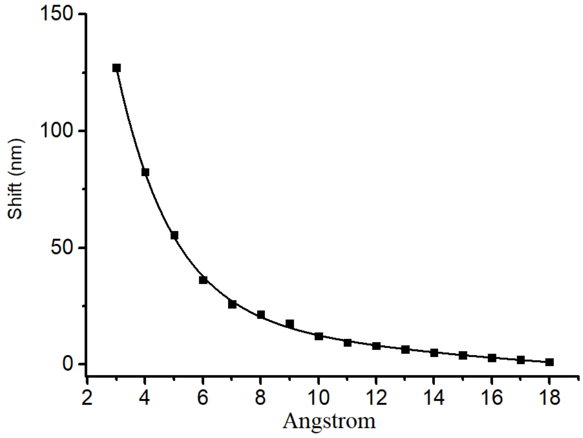

d) Reference atom C7

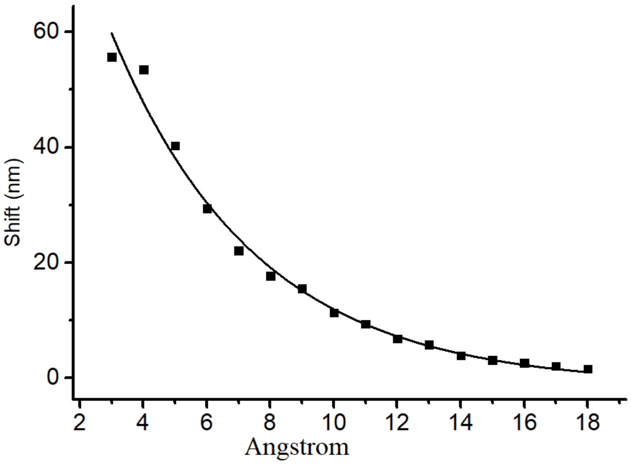

e) Reference atom C8

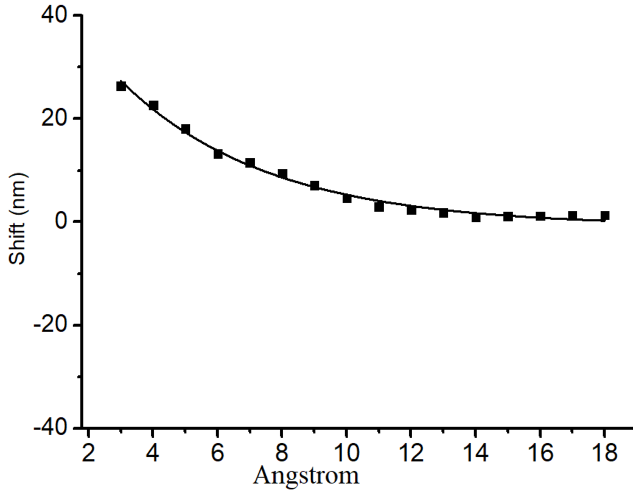

f) Reference atom C10

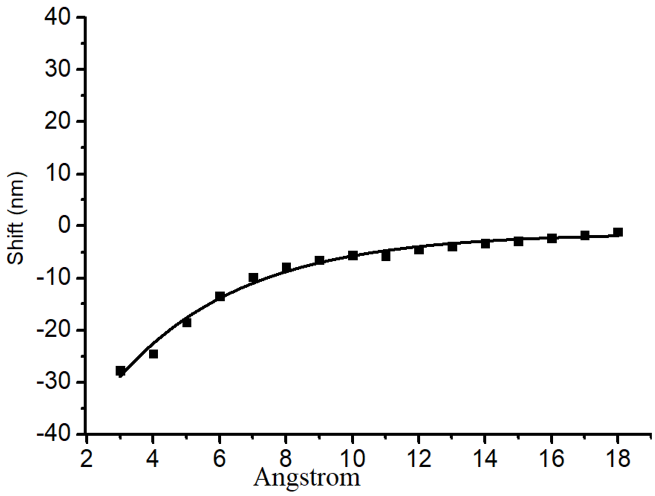

g) Reference atom C11

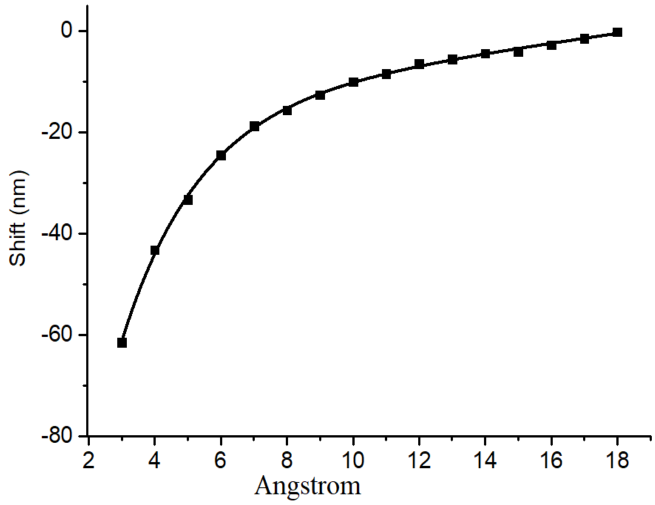

h) Reference atom C12

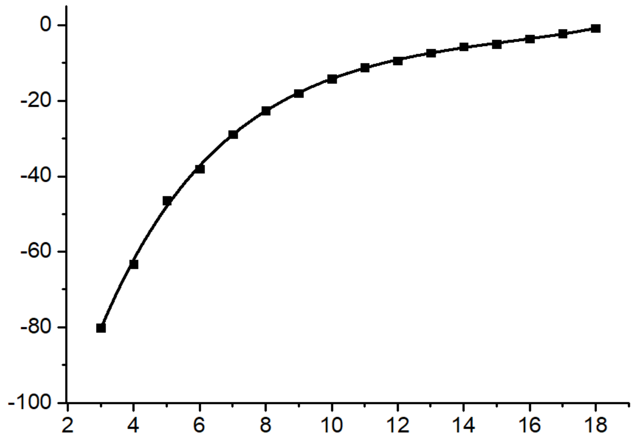

i) Reference atom C13

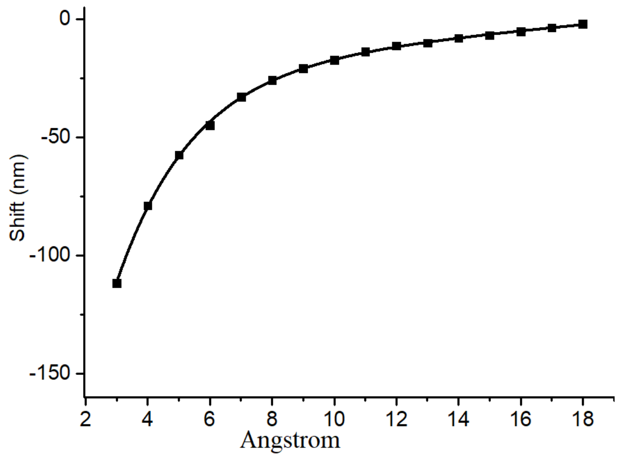

j) Reference atom C14

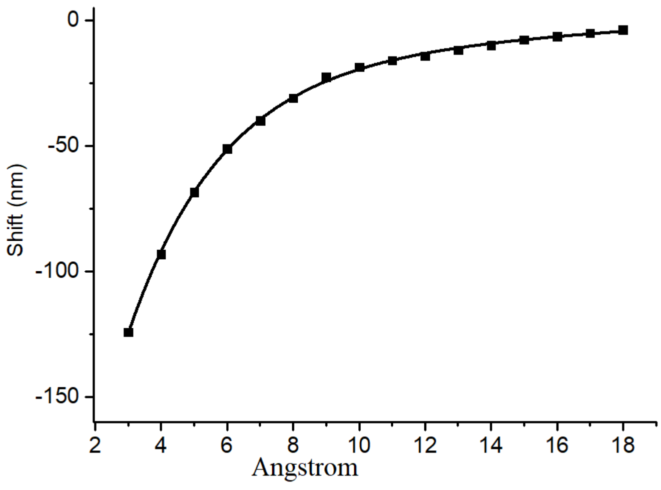

k) Reference atom C15

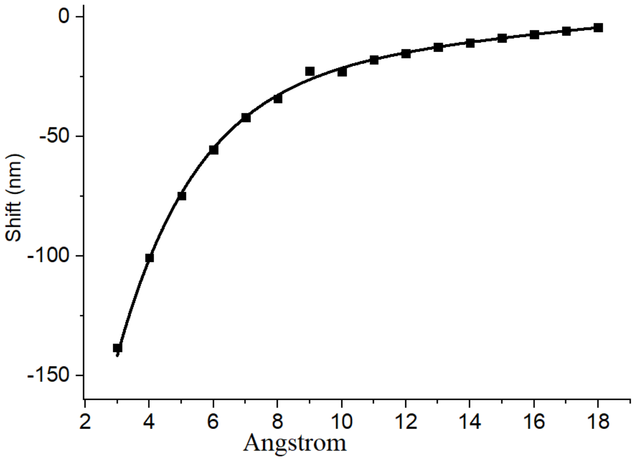

k) Reference atom N16

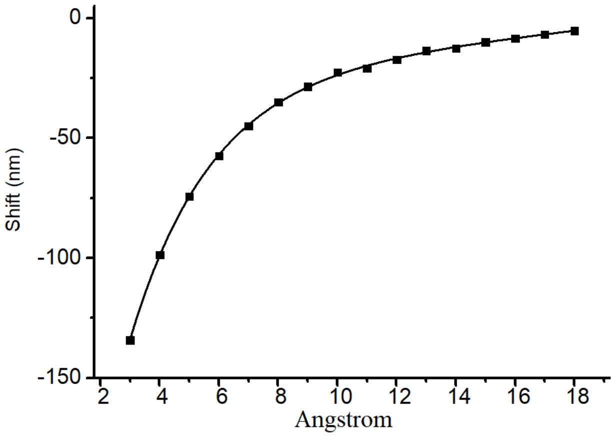

Figure S1

Table S1

11-cis PSB		Distance from the chromophore (Angstrom)															
Positive charge		3	4	5	6	7	8	9	10	11	12	13	14	15	16	17	18
Reference	C4	-108.3	-84.5	-61.4	-44.6	-31.2	-23.2	-15.4	-10.3	-8.1	-5.9	-5.6	-3.8	-2.2	-0.8	-0.2	0.4
atoms	C5	-119.7	-86.0	-60.7	-41.5	-27.5	-23.6	-19.9	-14.8	-11.3	-7.9	-7.1	-5.5	-4.0	-2.7	-0.3	-1.6
	C6	-115.2	-85.5	-59.1	-37.9	-21.4	-22.4	-18.7	-14.6	-11.1	-7.9	-7.0	-5.5	-4.2	-3.0	-2.5	-1.9
	C7	-112.4	-88.5	-54.6	-32.8	-20.2	-18.9	-16.1	-11.5	-8.7	-6.4	-5.7	-4.4	-3.3	-2.3	-1.7	-1.4
	C8	-70.5	-46.2	-31.4	-20.4	-14.6	-9.7	-6.9	-5.4	-4.6	-3.3	-3.2	-2.3	-1.6	-1.0	-0.7	-0.5
	C9	-17.3	-11.7	-10.0	-6.8	-3.4	-3.1	-2.2	-2.1	-2.2	-1.8	-1.6	-1.3	-1.1	-0.9	-0.9	-0.8
	C10	36.7	28.0	19.4	16.2	10.7	10.6	7.3	4.7	4.2	3.5	2.8	2.1	1.6	1.1	0.9	0.7
	C11	85.4	60.5	41.4	29.4	22.0	16.7	13.3	9.8	7.7	4.7	4.0	3.8	2.8	1.9	1.5	1.1
	C12	102.7	80.1	58.8	42.5	31.3	23.8	18.6	15.2	10.7	9.1	6.3	5.6	4.1	2.8	2.2	1.6
	C13	95.2	89.1	64.7	47.3	34.3	27.1	21.5	17.9	12.7	9.3	8.4	6.5	4.9	3.4	2.8	2.1
	C14	88.3	80.8	64.7	53.2	42.1	31.8	25.9	20.1	16.4	13.4	9.4	7.3	5.4	3.7	3.0	2.3
	C15	76.7	70.8	59.4	52.6	41.7	32.9	28.6	21.6	18.4	15.1	9.9	7.8	6.0	4.3	3.7	2.9
	N16	71.3	63.0	54.5	50.4	31.8	32.1	28.4	23.6	19.9	15.3	10.6	8.6	6.8	5.3	4.6	3.9

Table S2

11-cis PSB		Distance from the chromophore (Angstrom)															
Negative charge		3	4	5	6	7	8	9	10	11	12	13	14	15	16	17	18
Reference	C4	127.4	100.7	71.0	51.5	33.5	22.1	16.2	10.4	8.8	6.2	4.0	3.2	1.3	0.3	-0.8	1.4
atoms	C5	134.9	96.5	64.3	42.8	29.6	23.7	18.7	13.2	9.3	7.1	5.2	5.1	2.5	1.4	0.4	1.6
	C6	127.2	82.5	55.6	36.3	26.0	21.6	17.7	12.7	8.7	7.0	5.5	4.8	2.9	2.1	1.3	1.6
	C7	55.7	53.5	40.3	29.4	22.1	17.7	15.5	9.9	6.9	5.8	4.7	3.2	2.7	2.2	1.7	1.4
	C8	26.4	22.7	18.1	13.3	11.5	9.5	7.1	5.6	4.1	3.7	3.1	1.5	1.8	1.7	1.6	1.1
	C9	-13.8	-0.7	1.0	1.4	2.1	3.2	3.1	0.5	0.6	0.8	0.7	-0.4	0.4	0.7	1.0	0.7
	C10	-27.7	-24.5	-18.5	-13.4	-9.8	-7.9	-6.5	-4.7	-3.4	-2.5	-2.2	-2.3	-1.5	-0.7	0.0	0.2
	C11	-61.5	-43.2	-33.3	-24.5	-18.7	-15.6	-12.6	-9.6	-7.5	-6.1	-5.3	-4.3	-3.5	-2.4	-1.3	-0.5
	C12	-80.2	-63.3	-46.4	-38.1	-28.9	-22.6	-18.0	-13.9	-11.4	-9.4	-8.1	-6.2	-5.5	-4.1	-2.7	-1.3
	C13	-111.7	-78.9	-57.4	-44.9	-32.8	-25.8	-20.8	-17.4	-14.7	-12.4	-10.5	-8.0	-7.2	-5.7	-4.1	-2.2
	C14	-124.2	-93.2	-68.4	-51.0	-39.9	-30.8	-22.5	-20.0	-17.3	-14.6	-12.2	-9.6	-8.5	-6.9	-5.3	-3.2
	C15	-138.4	-100.7	-74.8	-55.5	-41.9	-34.1	-22.5	-21.7	-18.8	-15.9	-12.9	-11.1	-9.1	-7.6	-6.0	-4.2
	N16	-134.2	-98.7	-74.3	-57.4	-45.0	-35.0	-28.6	-22.7	-19.2	-16.1	-12.6	-12.3	-9.1	-7.7	-6.3	-5.3

Table S3

all-trans PSB		Distance from the chromophore (Angstrom)															
Positive charge		3	4	5	6	7	8	9	10	11	12	13	14	15	16	17	18
Reference	C4	-95.5	-75.5	-57.4	-44.0	-34.9	-26.9	-21.0	-16.5	-10.8	-7.8	-6.1	-4.5	-3.2	-1.9	-1.5	-0.9
atoms	C5	-105.5	-79.7	-58.5	-43.1	-32.7	-24.6	-18.3	-15.1	-9.4	-7.6	-6.0	-4.5	-3.2	-2.0	-1.5	-1.0
	C6	-103.1	-76.3	-53.6	-39.1	-28.0	-21.1	-15.2	-12.3	-7.5	-7.3	-6.2	-4.6	-3.2	-2.0	-1.4	-0.9
	C7	-89.3	-64.2	-42.4	-31.0	-21.5	-16.3	-11.6	-8.7	-6.5	-5.4	-4.7	-3.4	-2.3	-1.4	-0.9	-0.7
	C8	-60.8	-39.8	-23.5	-16.6	-11.8	-8.0	-5.9	-4.5	-3.7	-3.1	-2.7	-2.1	-1.5	-1.0	-0.8	-0.6
	C9	-23.7	-13.1	-5.7	-3.1	-2.4	-0.1	-0.5	-1.0	-0.6	-1.7	-0.7	-0.1	0.1	0.0	0.0	0.0
	C10	19.7	16.9	14.6	11.0	8.2	7.8	5.3	3.2	2.3	2.6	2.1	1.7	1.3	1.0	0.8	0.7
	C11	55.5	43.5	33.3	24.7	17.5	15.1	10.3	7.3	4.8	4.7	3.8	2.9	2.2	1.5	1.1	0.9
	C12	80.4	61.3	45.3	33.5	24.1	20.0	14.0	10.2	6.7	6.5	5.1	4.0	3.0	2.1	1.5	1.3
	C13	101.2	76.1	55.5	41.2	30.3	23.6	17.1	12.7	8.3	7.6	6.2	4.9	3.9	2.9	2.5	2.1
	C14	107.0	80.3	58.5	43.6	32.8	25.9	19.4	14.8	9.6	8.2	6.9	5.7	4.7	3.9	3.4	3.1
	C15	88.0	67.3	50.7	39.3	33.7	26.6	20.8	16.4	10.4	8.3	7.1	6.1	5.1	4.3	3.9	3.6
	N16	80.4	62.0	47.3	37.0	32.8	25.7	21.3	17.3	10.7	8.1	6.9	5.9	5.0	4.2	3.8	3.5

Table S4

all-trans PSB		Distance from the chromophore (Angstrom)															
Negative charge		3	4	5	6	7	8	9	10	11	12	13	14	15	16	17	18
Reference	C4	105.1	73.0	49.0	38.3	26.6	21.6	17.0	9.2	9.0	5.2	5.9	4.8	3.8	3.0	2.5	2.2
atoms	C5	100.3	70.4	51.1	35.4	28.2	23.4	17.1	12.7	8.9	6.7	5.4	4.4	3.6	2.8	2.5	2.2
	C6	78.0	58.0	39.0	28.8	22.4	19.0	13.4	8.8	7.5	5.0	4.8	4.0	3.3	2.7	2.4	2.2
	C7	53.3	37.7	21.2	17.7	15.3	13.5	9.2	5.7	4.9	3.1	3.5	3.0	2.5	2.1	2.0	1.8
	C8	30.4	23.2	16.3	8.8	7.4	6.3	4.5	2.4	2.2	1.0	1.6	1.4	1.3	1.1	1.0	1.0
	C9	4.8	-3.5	-2.2	-3.6	-0.9	0.6	-0.3	-1.0	-0.7	-1.0	-1.1	-1.0	-0.9	-0.9	-0.8	-0.8
	C10	-28.0	-19.7	-15.7	-12.7	-9.5	-6.0	-5.3	-4.7	-3.6	-3.2	-2.7	-2.2	-1.8	-1.4	-1.2	-1.1
	C11	-44.5	-35.8	-26.7	-21.0	-16.6	-12.5	-9.8	-7.1	-6.6	-4.7	-3.8	-3.0	-2.2	-1.6	-1.2	-1.0
	C12	-70.6	-52.7	-38.0	-29.6	-23.4	-17.9	-13.9	-10.1	-8.0	-6.5	-5.1	-3.9	-2.9	-2.0	-1.5	-1.2
	C13	-95.0	-67.1	-48.9	-37.7	-29.5	-23.0	-17.5	-13.9	-10.4	-8.5	-6.7	-5.2	-3.9	-2.7	-2.0	-1.7
	C14	-110.8	-78.5	-56.6	-43.1	-33.4	-26.0	-20.3	-15.3	-13.3	-9.4	-7.4	-5.6	-4.1	-2.8	-2.1	-1.6
	C15	-119.7	-86.1	-62.1	-46.4	-35.9	-27.9	-22.2	-17.1	-15.1	-10.3	-8.1	-6.3	-4.7	-3.2	-2.4	-1.9
	N16	-117.2	-83.7	-62.3	-46.3	-35.7	-28.7	-22.9	-18.3	-15.0	-10.7	-8.6	-6.7	-5.1	-3.7	-3.1	-2.4

Table S5

11-cis, dipole	N16				C12				C8				C4			
γ, degrees	3.5 Å	4.5 Å	5.5 Å	6.5 Å	3.5 Å	4.5 Å	5.5 Å	6.5 Å	3.5 Å	4.5 Å	5.5 Å	6.5 Å	3.5 Å	4.5 Å	5.5 Å	6.5 Å
0	1.7	-0.8	0.1	-0.8	-4.9	-4.3	-2.9	-2.4	-13.7	-9.5	-7.0	-5.2	-5.2	-1.4	-0.6	-0.3
30	7.5	5.0	2.3	1.2	-0.3	-1.2	-0.4	-0.3	-15.1	-8.8	-7.3	-5.0	-12.0	-7.4	-5.2	-2.8
60	11.9	7.2	4.5	2.3	4.6	2.7	2.4	1.6	-13.6	-8.6	-5.6	-3.8	-17.5	-10.4	-6.9	-5.2
90	12.4	7.3	5.2	3.1	8.6	6.5	5.6	3.5	-10.3	-5.1	-2.8	-1.7	-17.2	-11.1	-8.1	-5.2
120	8.5	6.0	3.7	2.1	10.1	7.6	5.8	3.6	-1.6	1.4	-0.1	0.1	-11.3	-7.7	-6.3	-4.9
150	2.8	1.6	0.9	1.3	7.6	6.9	4.6	3.3	8.0	4.3	4.1	3.3	-4.2	-3.8	-4.0	-2.7
180	-4.3	-2.8	-3.0	-1.9	5.4	3.2	2.1	1.8	13.0	7.6	5.1	3.5	2.5	1.6	0.6	-0.2
210	-9.4	-5.6	-4.7	-3.9	-2.1	-0.4	-0.7	-0.5	14.9	9.2	5.9	3.7	11.5	5.3	3.2	1.4
240	-13.2	-9.0	-5.0	-3.9	-7.1	-5.7	-3.9	-3.0	13.5	6.6	5.3	2.8	16.5	10.0	5.7	3.8
270	-13.9	-9.2	-4.8	-3.4	-11.4	-8.6	-7.0	-4.9	7.5	4.1	1.4	0.4	16.5	10.1	6.6	5.0
300	-8.7	-8.0	-4.8	-3.2	-12.7	-9.0	-7.5	-5.5	-0.3	-1.8	-1.1	-2.0	11.2	8.7	5.4	3.7
330	-4.5	-3.6	-2.7	-2.0	-9.8	-7.6	-5.3	-4.7	-7.8	-6.9	-5.5	-4.6	2.9	3.7	2.2	2.2
360	1.3	1.3	0.5	-0.8	-4.8	-3.1	-2.8	-2.4	-13.1	-9.4	-6.9	-5.2	-4.6	-2.1	-1.2	-0.3

Table S6

all-trans, dipole	N16				C12				C8				C4			
γ, degrees	3.5 Å	4.5 Å	5.5 Å	6.5 Å	3.5 Å	4.5 Å	5.5 Å	6.5 Å	3.5 Å	4.5 Å	5.5 Å	6.5 Å	3.5 Å	4.5 Å	5.5 Å	6.5 Å
0	2.9	1.7	1.3	-0.4	-4.9	-4.5	-2.5	-2.3	-8.9	-6.0	-3.4	-2.6	5.0	1.9	-0.3	-0.2
30	4.7	3.2	2.2	2.9	0.1	-1.2	-1.1	0.1	-9.8	-5.5	-4.1	-2.8	-2.4	-2.5	-2.4	-1.9
60	7.2	3.9	2.5	1.4	3.4	3.4	3.6	2.4	-9.2	-4.9	-3.3	-2.7	-6.8	-5.1	-3.4	-1.9
90	5.7	3.6	2.3	1.2	7.6	5.9	4.6	3.5	-3.7	-3.4	-2.5	-1.2	-10.8	-7.0	-4.1	-2.6
120	3.6	2.6	0.9	0.9	8.5	8.0	4.6	2.8	0.7	2.0	1.0	0.5	-10.3	-7.2	-3.5	-2.4
150	0.5	-0.7	-0.6	-0.4	8.1	5.1	3.6	1.7	4.4	3.5	3.3	2.7	-10.0	-6.0	-2.5	-1.5
180	-2.5	-1.1	-1.1	-0.5	4.8	2.1	0.5	0.5	8.0	6.0	3.6	2.7	-5.0	-2.6	-2.0	-0.2
210	-5.1	-4.1	-2.4	-1.8	-3.0	-1.9	-0.8	-0.4	10.3	5.8	3.2	3.0	2.7	2.4	1.7	0.8
240	-8.3	-5.5	-3.4	-1.8	-6.7	-4.5	-2.6	-1.5	7.0	4.4	1.4	2.0	7.9	5.5	3.0	2.0
270	-8.3	-6.1	-3.3	-1.2	-10.8	-6.6	-4.0	-2.0	3.8	0.9	1.5	0.2	10.8	7.6	4.1	2.5
300	-3.7	-3.7	-2.9	-2.5	-11.3	-6.5	-4.7	-3.0	0.0	-2.3	-2.1	-0.6	9.8	5.9	3.4	2.3
330	-0.5	-1.5	-2.0	-1.6	-8.6	-5.7	-4.4	-2.7	-3.3	-3.7	-2.6	-2.7	8.3	2.7	2.3	2.1
360	2.7	2.7	0.8	0.2	-3.9	-4.1	-2.6	-1.1	-7.6	-6.3	-3.7	-2.2	3.5	1.1	0.3	-0.4

The verification of the 'cylindrical symmetry' assumption.

To confirm that the impact of a charged/polar residue to λmax depends only on its charge/dipole moment and its distance to/orientation along the chromophore axis but not a radial angle, we performed the following set of calculations.

We placed a unit negative charge at 4 Å from the C4 atom of the PSB, and calculated the corresponding Δλmax (0º). Then we rotated the charge around the PSB axis, keeping the distance from the C4 atom fixed, and calculated the Δλmax values for radial angles 60º, 120º, 180º. Then we calculated the corresponding differences: ΔΔλmax (60º) = Δλmax (60º) - Δλmax (0º); ΔΔλmax (120º) = Δλmax (120º) - Δλmax (0º); ΔΔλmax (180º) = Δλmax (180º) - Δλmax (0º). The same set of calculations was performed for the C6, C10, N16 reference atoms. The results are presented in **Table S7**.

Table S7.

ΔΔλmax// Ref. atom	C4	C6	C10	N16
ΔΔλmax (60º)	3.6 nm	1.8 nm	0.0 nm	0.0 nm
ΔΔλmax (120º)	3.5 nm	-0.5 nm	0.0 nm	0.0 nm
ΔΔλmax (180º)	2.5 nm	1.3 nm	0.0 nm	0.0 nm
